# Role of CT texture features for predicting outcome of pancreatic cancer patients with liver metastases

**DOI:** 10.7150/jca.49569

**Published:** 2021-02-22

**Authors:** Junjie Hang, Kequn Xu, Ruohan Yin, Yueting Shao, Muhan Liu, Haifeng Shi, Xiaoyong Wang, Lixia Wu

**Affiliations:** 1 Department of Oncology, Changzhou No.2 People's Hospital, Nanjing Medical University, Xinglong Road 19, Changzhou 213000, China.; 2 Department of Medical Imaging, Changzhou No.2 People's Hospital, Nanjing Medical University, Xinglong Road 19, Changzhou 213000, China.; 3 Department of Gastroenterology, Changzhou No.2 People's Hospital, Nanjing Medical University, Xinglong Road 19, Changzhou 213000, China.; 4 Department of Oncology, Shanghai JingAn District ZhaBei Central Hospital, Zhonghuaxin Road 619, Shanghai 200040, China.

**Keywords:** CT texture features, liver metastases, pancreatic cancer, prognostic nomogram, radiomics score

## Abstract

**Objective:** The purpose of this study was to evaluate the prognostic value of computed tomography (CT) texture features of pancreatic cancer with liver metastases.

**Methods:** We included 39 patients with metastatic pancreatic cancer (MPC) with liver metastases and performed texture analysis on primary tumors and metastases. The correlations between texture parameters were assessed using Pearson's correlation. Univariate Cox proportional hazards model was used to assess the correlations between clinicopathological characteristics, texture features and overall survival (OS). The univariate Cox regression model revealed four texture features potentially correlated with OS (P<0.1). A radiomics score (RS) was determined using a sequential combination of four texture features with potential prognostic value that were weighted according to their β-coefficients. Furthermore, all variables with P<0.1 were included in the multivariate analysis. A nomogram,which was developed to predict OS according to independent prognostic factors, was internally validated using the C-index and calibration plots. Kaplan-Meier analysis and the log-rank test were performed to stratify OS according to the RS and nomogram total points (NTP).

**Results:** Few significant correlations were found between texture features of primary tumors and those of liver metastases. However, texture features within primary tumors or liver metastases were significantly associated. Multivariate analysis showed that Eastern Cooperative Oncology Group performance status (ECOG PS), chemotherapy, Carbohydrate antigen 19-9 (CA19-9), and the RS were independent prognostic factors (P<0.05). The nomogram incorporating these factors showed good discriminative ability (C-index = 0.754). RS and NTP stratified patients into two potential risk groups (P<0.01).

**Conclusion:** The RS derived from significant texture features of primary tumors and metastases shows promise as a prognostic biomarker of OS of patients with MPC. A nomogram based on the RS and other independent prognostic clinicopathological factors accurately predicts OS.

## Introduction

Pancreatic cancer is the seventh leading cause of cancer-related death worldwide, and its incidence closely parallels mortality 1. Approximately 53% of patients with pancreatic cancer present with metastatic disease upon initial diagnosis 2. The liver is one of the most common metastatic sites of pancreatic cancer, and patients with MPC with liver metastases experience much poorer prognosis than those with other metastatic patterns 3. Although a small portion of patients with MPC can benefit in OS from targeted therapy and immunotherapy, cytotoxic chemotherapy remains the mainstay of treatment 4. As we move towards an era of precision medicine, more information about the tumor facilitates decision-making that will improve patients' outcomes.

CT is routinely used in the diagnosis and efficacy evaluation of pancreatic cancer with size-based or shape-based measures of the tumor 5, 6. However, the information acquired from CT is limited, because it mainly relies upon visual evaluations. Fortunately, recent research shows that the interpretations of CT images are augmented by texture analysis, which potentially reveals the underlying tumor biology 7. Texture analysis of CT involves a computational process in which weakening CT levels among image voxels are spatially quantified to associate structural features of tumors with pixel variability 8. The histogram-based method and spatial arrangement-based methods are most frequently used for texture analysis, and the corresponding texture features are evaluated using first-order statistics and higher-order statistics 9. These methods are applied for detection, characterization, and monitoring of tumors 7, 10.

The texture features of pancreatic cancer facilitate diagnosis and treatmet effect prediction. For example, texture analysis differentiates pancreatic adenocarcinoma from other pancreatic lesions such as pancreatic neuroendocrine carcinoma, mass-forming pancreatitis, and pancreatic lymphoma 11-13. Certain CT texture features, such as tumor dissimilarity and kurtosis, were significantly correlated with OS of patients with pancreatic cancer 14-17. Furthermore, Marc A. Attiyeh et al. found CT texture features were associated with SMAD4 status and stromal content in pancreatic cancer 18. In addition, texture features may play a role in predicting the response of patients to chemotherapy 19, 20. However, to the best of our knowledge, there is paucity of studies about the potential prognostic value of CT texture features of primary tumors and liver metastases of pancreatic cancer. The aim of this study therefore was to determine their value in predicting the survival of MPC patients with liver metastases.

## Methods

### Patients

39 consecutive MPC patients with liver metastases treated at Changzhou No. 2 People's Hospital between January 2016 and June 2019 were retrospectively enrolled in this study. The inclusion criteria were as follows: (1) newly diagnosed, and pathologically confirmed pancreatic adenocarcinoma, (2) absence of concurrent cancer at another site, and (3) availability of complete records of baseline clinicopathological features. Demographic and clinicopathological features were collected from the electronic medical record. Informed consent was obtained from each patient and ethical approval was obtained by the Ethics Committee of Changzhou No.2 People's Hospital.

### CT image acquisition

Contrast-enhanced CT examinations were performed using a 128-row dual-source CT scanner (SOMATOM Definition Flash, Siemens, Germany) at 120 kV, tube current modulation, and 1 mm reconstructed section thickness. All patients were instructed to fast for at least 8 h before the administration of intravenous contrast (Iohexol, 300 mg/ml, 80 ml, at a rate of 3 ml/s). After the injection of contrast agent, patients were subsequently subjected to double-helical scanning during the arterial and portal venous phases. The region of interest (ROI) was selected during the portal venous phase, because margins of pancreatic cancers are most consistently detected during in this phase.

### Image processing

ROIs were drawn on each slice of the primary pancreatic cancer and the liver metastases, using the Semantic Segmentation Editor. ROIs were subsequently extracted for texture analysis using Local Image Features Extraction (LIFEx, version 5.10, https://www.lifexsoft.org/). In the segmented tumors, the volume of interest (VOI) and histogram were calculated as first-order features. For calculations of second- and high-order texture features, the number of grey levels used to resample the ROI content was set to 64.0 as described in previous studies. The Cartesian coordinates for spatial resampling were 2.0 mm (X-direction), 2.0 mm (Y-direction), and 1.0 mm (Z-direction). Texture features were evaluated using four texture matrices, including the grey-level co-occurrence matrix (GLCM), the grey-level run length matrix (GLRLM), the neighborhood grey-level different matrix (NGLDM), and the grey-level zone length matrix (GLZLM). The texture features of the largest cross-section of each tumor and its corresponding liver metastases were used for further analysis.

### Statistical analysis

Statistical analysis was conducted using R software (version 3.6.1, Institute for Statistics and Mathematics, Vienna, Austria). The correlations between texture variables were assessed using Pearson's correlation coefficient with the R package “psych”. A univariate Cox regression model was used to assess the correlations between clinicopathological characteristics, texture features, and OS. A radiomics score (RS) was calculated according to the results of univariate analysis, where RS = 0.280 × tGLZLM_LZE + 0.337 × tGLZLM_LZHGE + 0.334 × lKurtosis + 0.364 × lNGLDM_Busyness. In this formula, each variable was weighted using its β-coefficient derived from the Cox regression model. Variables with P<0.1 were included in the multivariate analysis. A nomogram was developed to predict OS according to independent prognostic factors using the R package “rms”. The internal validation of the nomogram was performed using the C-index and a calibration plot generated using bootstrapping with 1000 resamples. Kaplan Meier analysis and the log-rank test were performed to stratify OS according to groups of RS and nomogram total points (NTP).

## Results

### Patients' characteristics

The baseline clinicopathological characteristics of patients with MPC are listed in Table 1. The patients included 16 (41%) women and 23 (59%) men, and 79.5% (n=31) of them had ECOG PS = 2 or 3, and 8 (20.5%) had ECOG PS = 1. Among the 23 (59.0%) patients with MPC, the lesions were in the body and tail of the pancreas, whereas among 16 (41.0%) patients, the lesions were in the head and neck. Furthermore, 9 (23.1%) patients had one site of liver metastasis, and 30 (76.9%) patients had >1 site. The mean of the longest diameters of liver metastases was 3.1 cm (SD = 2.7). Among all patients, 14 (35.9%) received combination therapy, 13 (33.3%) received monotherapy, and 12 (30.8%) patients received only best support care (BSC).

### Texture analysis

The mean values and standard deviations of all texture features are listed in Supplementary Table 1, and the values of these features were normalized for further analysis.

**Figure 1:**
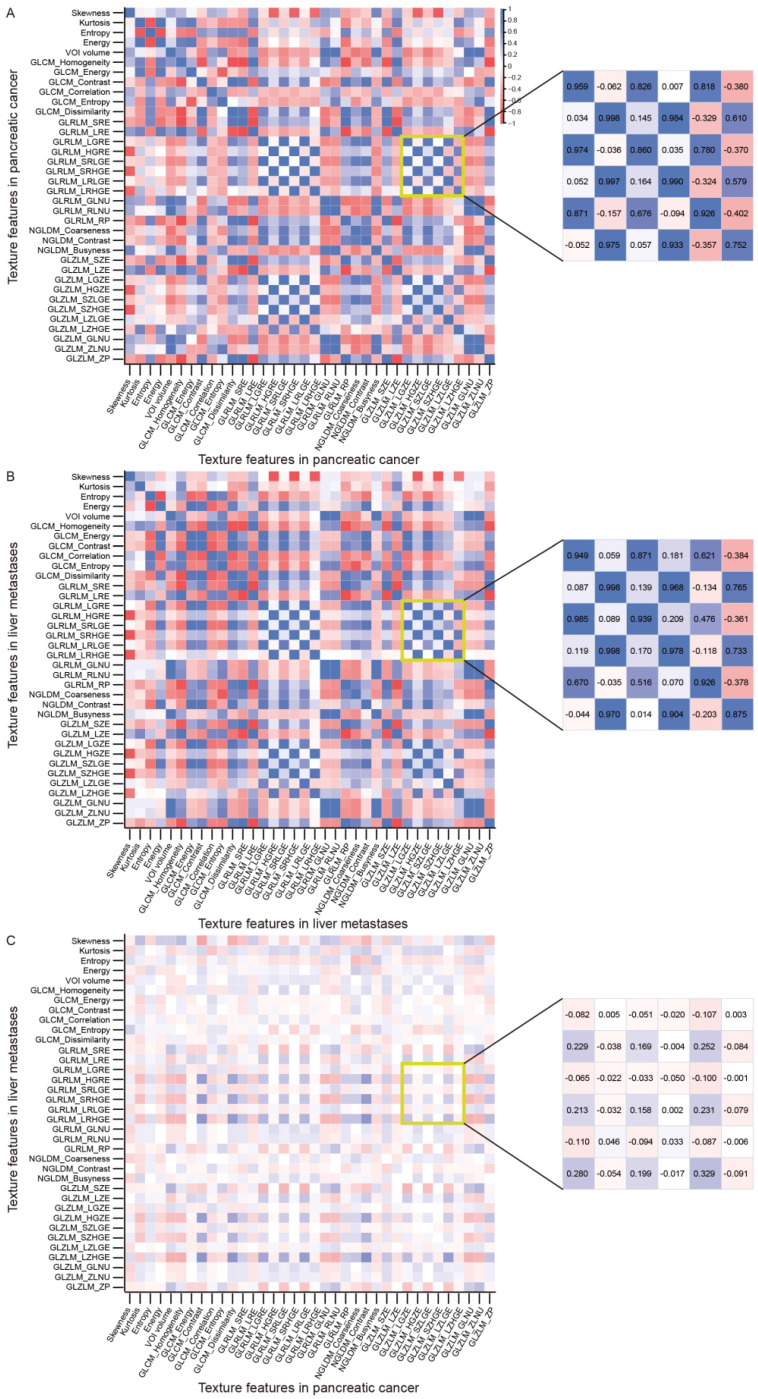
The correlations between texture parameters. The correlations between texture parameters within primary pancreatic cancer (A) or within liver metastases (B) were relatively significant (mean r = 0.444), and some had similar correlation patterns (like those within the yellow box). However, few significant correlations were found between texture features of the primary tumor and those of liver metastases (mean r = 0.119, C).

Pearson's correlation coefficient was used to investigate the correlations between texture features listed in Supplementary Table 2 to 4. Intriguingly, we found that the texture features within the primary tumor (mean r = 0.444, Figure 1A) or liver metastases (mean r = 0.444, Figure and 1B) significantly correlated with each other, and some had similar correlation patterns (like those within the yellow box). However, few significant correlations were found between texture features of the primary tumor and those of liver metastases (mean r = 0.119, Figure 1C). For example, the GLZLM_LGZE of the primary tumor (tGLZLM_LGZE) was significantly correlated with the GLRLM_LGRE of the primary tumor (tGLRLM_LGRE) (r = 0.959). Likewise, the GLZLM_LGZE of the liver metastases (lGLZLM_LGZE) was also significantly correlated with the GLRLM_LGRE of the liver metastases (lGLRLM_LGRE) (r = 0.949). However, the correlation between tGLZLM_LGZE and lGLRLM_LGRE was not significant (r= -0.082). Such phenomenon can also be seen in other pairs such as GLRLM_HGRE and GLZLM_HGZE.

### The prognostic significance of the RS

In the univariate Cox regression model, four texture features were significantly correlated with OS (P<0.1, Table 2). The RS was determined using a sequential combination of these four features weighted according to their β-coefficients. Univariate analysis revealed that age, ECOG PS, weight loss, chemotherapy, CA19-9, the longest diameter of liver metastases, and the RS were correlated with OS. Furthermore, multivariate analysis showed that ECOG PS, chemotherapy, CA19-9, and the RS were independent prognostic factors for OS (each, P<0.05, Table 3).

**Figure 2:**
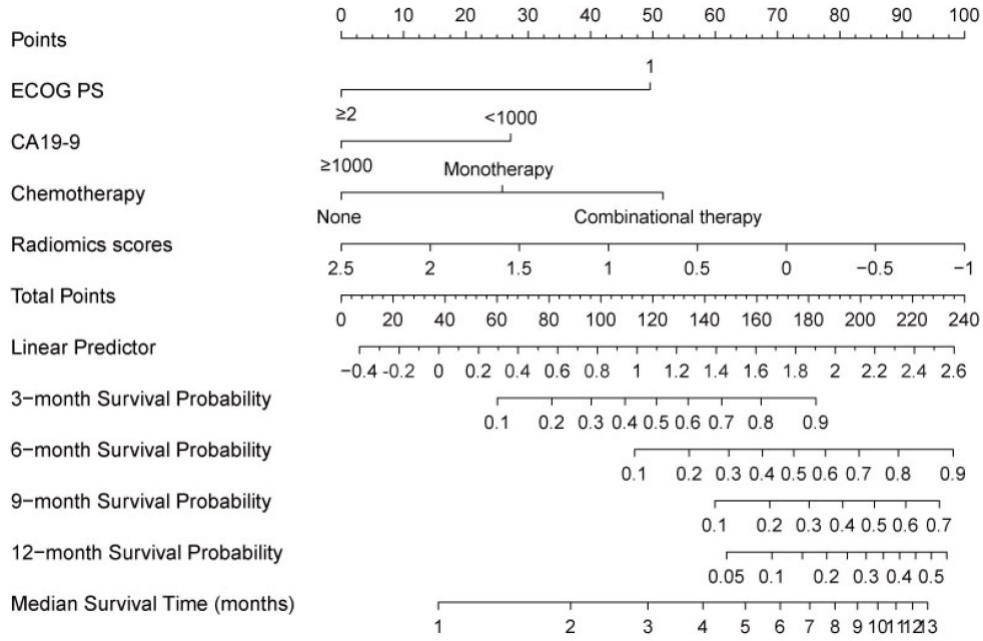
Prognostic nomogram for predicting 3-, 6-, 9-, and 12-month overall survival probability based on ECOG PS, liver metastases, CA19-9, and radiomics scores in pancreatic cancer patients with liver metastases. Each status of 4 independent factors has a corresponding value to the “Points” line at the top of the scale. Then the total point score was calculated by summing these 4 “Points” values. Based on the score, draw a downward vertical line from the “Total Points” line, predicting 3-, 6-, 9-, and 12- month survival probability in a given patient.

**Figure 3:**
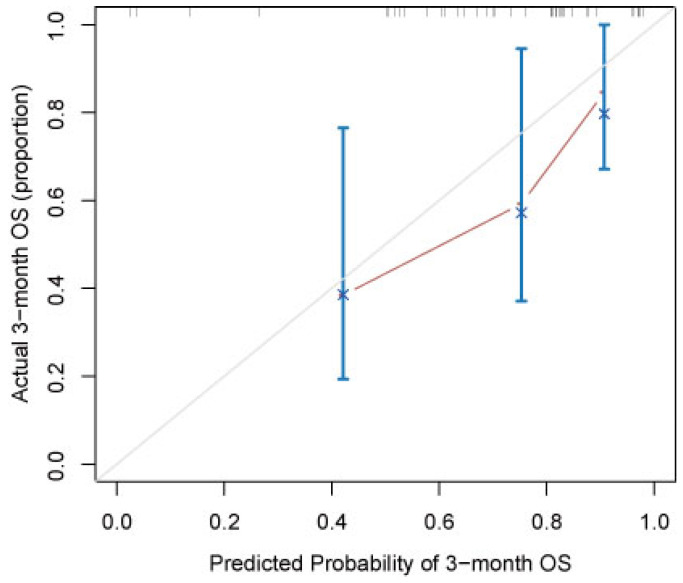
Calibration curves of the nomogram for the prediction of 3-month survival. The diagonal line: the ideal calibrated model. Black line: actual calibration. Circles: median. X: mean. 95% confidence intervals are depicted for each point along the calibration curve.

**Figure 4:**
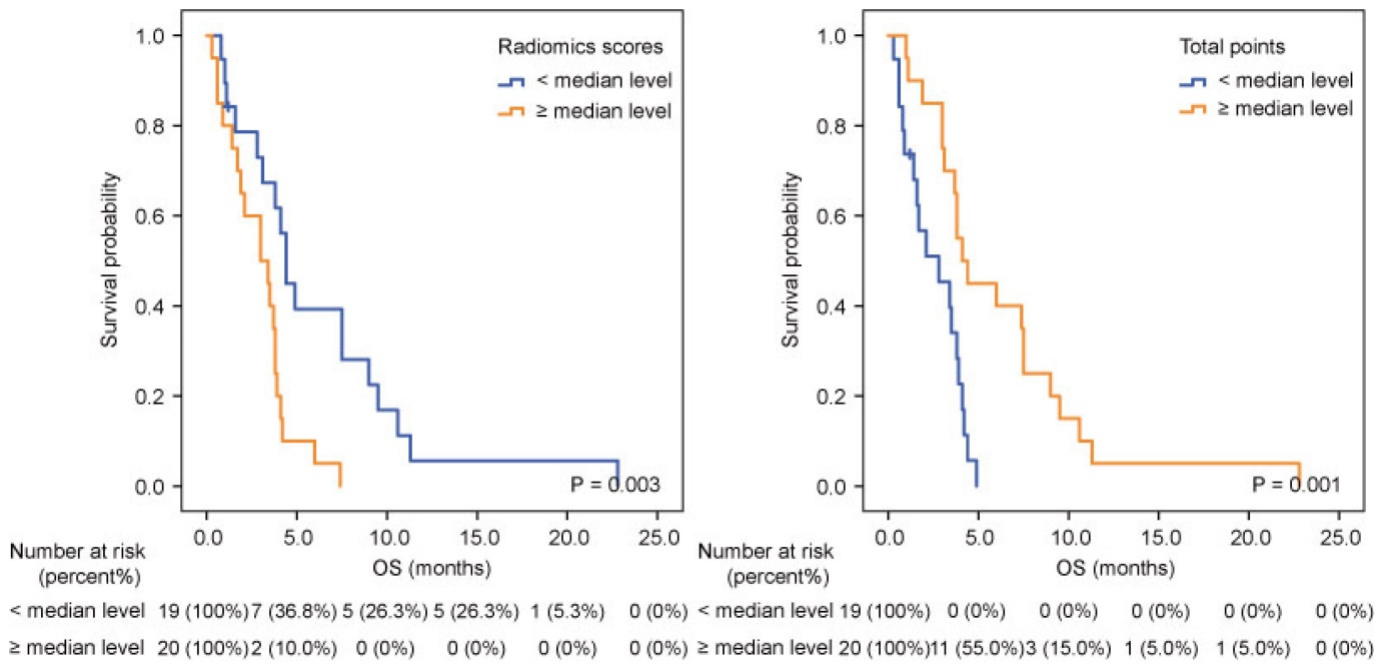
Kaplan-Meier analysis according to radiomics scores (A) and nomogram total points (B) in pancreatic cancer patients with liver metastases.

A nomogram developed incorporating these independent prognostic factors (Figure 2) predicted the median OS and survival probabilities of patients with MPC at 3-month, 6-months, 9-months and 12-months. The nomogram achieved good discriminative ability (C-index = 0.754), compared with that (C-index = 0.712) of the model that did not incorporate the RS. After adjustment using bootstrapping with respective 1000 re-samples, calibration plot was used to compare the predicted with actual survival probabilities (Figure 3). All patients were divided into two groups according to the median value of NTPs to stratify OS. Kaplan-Meier analysis showed that the median OS of patients with NTPs greater than the median level was 4.1 months (95%CI, 2.8-5.4), which was significantly higher compared with that of patients with NTPs below the median level (median OS = 2.8 months, P=0.001, Figure 4).

## Discussion

In this study, we used texture analysis to evaluate CT images of primary tumors and liver metastases of patients with MPC. We showed that the RS of significant texture features was an independent prognostic factors of OS, as revealed by unvariate analysis. A nomogram, which was based on RS and other independent prognostic clinicopathological factors, achieved good discriminative ability.

In univariate analysis, GLZLM_LZE and GLZLM_LZHGE of the primary tumor, as well as Kurtosis and NGLDM_Busyness of liver metastases, showed potential correlations with survival. Briefly, GLZLM reflects the size of homogeneous zones for each grey-level in three dimensions. LZE and LZHGE represent the distribution of the long homogeneous zones and its high grey-level subtypes, respectively. Furthermore, kurtosis reflects the shape of the grey-level distribution in the ROI. NGLDM_Busyness provides information on the spatial frequency of three-dimensional changes in intensities of grey-level between one voxel and its corresponding 26 neighbors. Generally, higher levels of these parameters reflect increased histological heterogeneity of the tumor and metastases, which will lead to poor prognosis of patients.

Several studies have reported the prognostic value of CT texture features for pancreatic cancer. For example, in patients with resectable pancreatic cancer patients, normalized dissimilarities and inverse difference serve as independent prognostic biomarkers of OS 14. Similarly, quantitation of imaging features could predict survival when combined with CA19-9 levels of patients with pancreatic cancer who undergo resection 21. Furthermore, the texture feature of CT images facilitates predicting OS in conjunction with the expression of HMGA2 and C-MYC in pancreatic cancer 22. More recent studies show that the prognosis of patients with locally advanced pancreatic cancer patients who undergo stereotactic body radiation, can be predicted according to the mean value, GLCM_Homogeneity, standard deviation value and GLCM_Dissimilarity of texture features 23. In patients with unresectable pancreatic cancer, Sandrasegaran et al found that the mean positive pixel value determined using a medium spatial filter was significantly associated with OS 15. In addition, Cheng et al found the tumor standard deviation was independently correlated with progression-free survival (PFS) and OS, while skewness was independently associated with PFS 16.

Here we show that the RS derived from the four aforementioned texture features was independently correlated with OS of patients with MPC with liver metastases. Furthermore, the nomogram, based on the RS, CA19-9, ECOG PS, and chemotherapy, achieved sufficient discriminative ability (C-index: 0.754), which was significantly higher compared with that of a model that did not incorporate RS (C-index: 0.712). These results suggest that the texture features of CT images play a pivotal role in predicting the outcome of patients with MPC with liver metastases.

The limitations of our study include its relatively small sample size and relatively wider range of texture features, which may introduce bias. Furthermore, patients received different types of chemotherapy or BSC. The heterogeneity of treatment strategies may influence the outcome, although we included this variable in multivariate analysis. Moreover, the use of the regions encompassing the largest cross-section of the tumor and its corresponding liver metastases may not comprehensively reflect the lesion, because of intrinsic tumor heterogeneity. Independent cohorts are therefore required to externally validate our results, along with the demonstration of the reproducibility and prognostic value of these texture features.

In conclusion, the RS based on texture features of the primary tumor and liver metastases shows promise as a prognostic imaging biomarker for patients with MPC with liver metastases. The nomogram developed here incorporating the RS and prognostic clinicopathological valuables will serve to accurately predict the outcomes of patients with pancreatic cancer.

## Supplementary Material

Supplementary tables.

## Figures and Tables

**Table 1:** Baseline clinicopathological characteristics of patients

Characteristics		
Age (year)	Mean ± SD	66.5±11.2
Gender	Male	23 (59.0%)
	Female	16 (41.0%)
ECOG PS	1	8 (20.5%)
	2 or 3	31 (79.5%)
Hypertension	Yes	16 (41.0%)
	No	23 (59.0%)
Diabetes	Yes	9 (23.1%)
	No	30 (76.9%)
Primary tumor location	Head and neck	16 (41.0%)
	Body and tail	23 (59.0%)
Abdominal pain	Yes	29 (74.4%)
	No	10 (25.6%)
Weight loss	Yes	6 (15.4%)
	No	33 (84.6%)
Chemotherapy	Combination therapy	14 (35.9%)
	Monotherapy	13 (33.3%)
	None	12 (30.8%)
CEA (ng/ml)	Mean ± SD	60.6±145.7
CA19-9 (U/ml)	Mean ± SD	556.9±440.1
Number of liver metastases	1	9 (23.1%)
	≥2	30 (76.9%)
Longest diameter of liver metastases	Mean ± SD	3.1±2.7

**Table 2:** Univariate analysis of prognostic texture features for OS

Characteristics	β	HR	95%CI	*P*
tGLZLM_LZE	0.280	1.324	0.954-1.836	0.093
tGLZLM_LZHGE	0.337	1.401	0.979-2.005	0.065
lKurtosis	0.334	1.396	0.992-1.964	0.055
lNGLDM_Busyness	0.364	1.439	1.028-2.013	0.034

**Table 3:** Univariate and multivariate analysis of prognostic factors for OS

Characteristics	Univariate analysis			Multivariate analysis		
	HR	95% CI	*P*	HR	95% CI	*P*
Age	1.027	0.998-1.057	0.073	1.028	0.992-1.066	0.126
**Gender**						
Male	0.830	0.426-1.619	0.586			
Female	Ref					
**ECOG PS**						
<2	0.149	0.044-0.503	0.002	0.251	0.064-0.985	0.048
≥2	Ref			Ref		
**Hypertension**						
Yes	1.112	0.799-1.548	0.529			
No	Ref					
**Diabetes**						
Yes	1.29	0.875-1.901	0.198			
No	Ref					
**Primary tumor location**						
Head and neck	1.046	0.542-2.017	0.894			
Body and tail	Ref					
**Abdominal pain**						
Yes	1.139	0.777-1.669	0.504			
No	Ref					
**Weight loss**						
Yes	2.671	1.083-6.592	0.033	1.032	0.324-3.283	0.957
No	Ref					
**Chemotherapy**						
Combination therapy	0.214	0.086-0.533	0.001	0.260	0.092-0.732	0.011
Monotherapy	0.284	0.116-0.696	0.006	0.206	0.075-0.562	0.002
None	Ref			Ref		
**CEA (ng/ml)**						
<5	1.262	0.648-2.461	0.494			
≥5	Ref					
**CA19-9 (U/ml)**						
<1000	0.330	0.152-0.714	0.005	0.310	0.124-0.775	0.012
≥1000	Ref			Ref		
**Number of liver metastases**						
1	0.773	0.357-1.675	0.514			
≥2	Ref					
Longest diameter of liver metastases	1.125	0.982-1.288	0.089	0.862	0.715-1.039	0.119
Radiomics score	2.453	1.483-4.055	<0.001	3.193	1.474-6.917	0.003

## Abbreviations

CT: computed tomography
MPC: metastatic pancreatic cancer
OS: overall survival
RS: radiomics score
NTP: nomogram total points
ECOG PS: Eastern Cooperative Oncology Group performance status
CA19-9: Carbohydrate antigen 19-9
ROI: region of interest
VOI: volume of interest
GLCM: grey-level co-occurrence matrix
GLRLM: grey-level run length matrix
NGLDM: neighborhood grey-level different matrix
GLZLM: grey-level zone length matrix
BSC: best support care
tGLZLM_LGZE: GLZLM_LGZE of the primary tumor
tGLRLM_LGRE: GLRLM_LGRE of the primary tumor
lGLZLM_LGZE: GLZLM_LGZE of the liver metastases
lGLRLM_LGRE: GLRLM_LGRE of the liver metastases
PFS: progression-free survival
